# *catena*-Poly[[tribenzyl­tin(IV)]-μ-(*E*)-3-phenyl­prop-2-enoato-κ^2^ *O*:*O*′]

**DOI:** 10.1107/S1600536811017247

**Published:** 2011-05-14

**Authors:** Kong Mun Lo, Seik Weng Ng

**Affiliations:** a Department of Chemistry, University of Malaya, 50603 Kuala Lumpur, Malaysia

## Abstract

The Sn^IV^ atom in the title carboxyl­ate-bridged polymer, [Sn(C_7_H_7_)_3_(C_9_H_7_O_2_)]_*n*_, exists in a *trans*-C_3_SnO_2_ trigonal–bipyramidal geometry (average covalent Sn—O = 2.167 Å, average dative Sn—O = 2.361 Å and average O—Sn—O = 169.6°). The polymer propagates as a helical chain along the *b* axis with a repeat distance that is half the *b*-axial length. There are four independent formula units in the asymmetric unit; two are disposed about a false center of inversion with respect to the other two so that the space group emulates a centric space group.

## Related literature

Trialkyl­tin(IV) carboxyl­ates generally contain five-coordinate Sn atoms and are carboxylate-bridged polymers; see: Ng *et al.* (1988 ▶). For the structure of tribenzyl­tin acetate, see: Ferguson *et al.* (1995 ▶). For the structure of tribenzyl­tin *p*-nitro­cinnamate, see: Thong *et al.* (2008 ▶). For the use of the Hooft and Flack parameters in confirming the *P*2_1_ space-group description, see: Hooft *et al.* (2008 ▶); Spek (2009 ▶). The polar *P*2_1_ space group is sometimes assigned incorrectly, see: Clemente & Marzotto (2003 ▶, 2004 ▶).
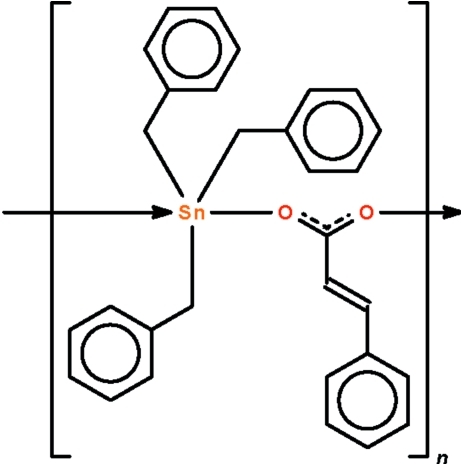


## Experimental

### 

#### Crystal data

[Sn(C_7_H_7_)_3_(C_9_H_7_O_2_)]
*M* *_r_* = 539.21
Monoclinic, 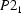

*a* = 19.5847 (3) Å
*b* = 10.2404 (2) Å
*c* = 26.6224 (4) Å
β = 110.2838 (8)°
*V* = 5008.15 (15) Å^3^
*Z* = 8
Mo *K*α radiation
μ = 1.04 mm^−1^
*T* = 100 K
0.20 × 0.10 × 0.10 mm

#### Data collection

Bruker SMART APEX diffractometer
Absorption correction: multi-scan (*SADABS*; Sheldrick, 1996 ▶) *T*_min_ = 0.818, *T*_max_ = 0.903
47886 measured reflections
22492 independent reflections
19107 reflections with *I* > 2σ(*I*)
*R*_int_ = 0.049

#### Refinement

*R*[*F* ^2^ > 2σ(*F* ^2^)] = 0.042
*wR*(*F* ^2^) = 0.087
*S* = 0.97
22492 reflections
1189 parameters
1 restraint
H-atom parameters constrained
Δρ_max_ = 0.83 e Å^−3^
Δρ_min_ = −0.79 e Å^−3^
Absolute structure: Flack (1983 ▶), 10328 Friedel pairs
Flack parameter: 0.00 (2)

### 

Data collection: *APEX2* (Bruker, 2009 ▶); cell refinement: *SAINT* (Bruker, 2009 ▶); data reduction: *SAINT*; program(s) used to solve structure: *SHELXS97* (Sheldrick, 2008 ▶); program(s) used to refine structure: *SHELXL97* (Sheldrick, 2008 ▶); molecular graphics: *X-SEED* (Barbour, 2001 ▶); software used to prepare material for publication: *publCIF* (Westrip, 2010 ▶).

## Supplementary Material

Crystal structure: contains datablocks global, I. DOI: 10.1107/S1600536811017247/si2355sup1.cif

Structure factors: contains datablocks I. DOI: 10.1107/S1600536811017247/si2355Isup2.hkl

Additional supplementary materials:  crystallographic information; 3D view; checkCIF report

## Figures and Tables

**Table:** 

Sn1—O1	2.165 (3)
Sn1—O2^i^	2.352 (4)
Sn2—O3	2.177 (3)
Sn2—O4^ii^	2.338 (3)
Sn3—O5	2.153 (3)
Sn3—O6^iii^	2.403 (4)
Sn4—O7	2.174 (3)
Sn4—O8^iv^	2.352 (4)

**Table:** 

O1—Sn1—O2^i^	171.2 (1)
O3—Sn2—O4^ii^	169.6 (1)
O5—Sn3—O6^iii^	168.4 (1)
O7—Sn4—O8^iv^	169.4 (1)

Symmetry codes: (i) 
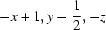
; (ii) 
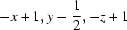
; (iii) 
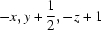
; (iv) 
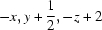
.

**Fig. 1.**
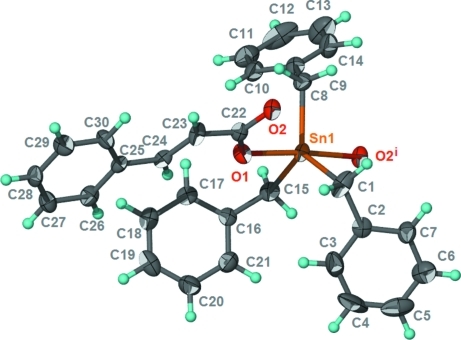
Thermal ellipsoid plot (Barbour, 2001) of a portion of the polymeric chain of Sn(C7H7)3(C8H7O2) at the 70% probability level (first formula unit); hydrogen atoms are drawn as spheres of arbitrary radius.

**Fig. 2.**
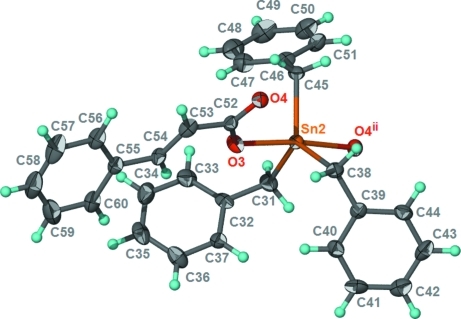
Thermal ellipsoid plot (Barbour, 2001) of a portion of the polymeric chain of Sn(C7H7)3(C8H7O2) at the 70% probability level (second formula unit); hydrogen atoms are drawn as spheres of arbitrary radius.

**Fig. 3.**
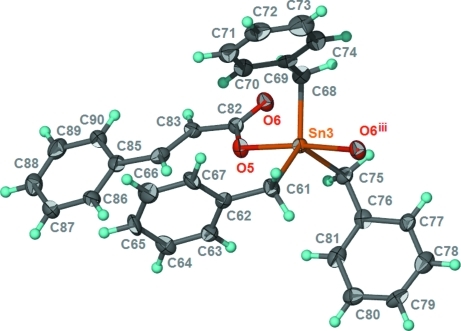
Thermal ellipsoid plot (Barbour, 2001) of a portion of the polymeric chain of Sn(C7H7)3(C8H7O2) at the 70% probability level (third formula unit); hydrogen atoms are drawn as spheres of arbitrary radius.

**Fig. 4.**
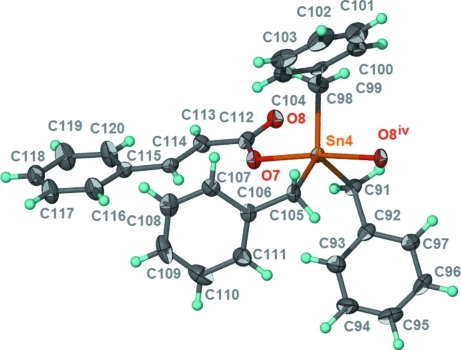
Thermal ellipsoid plot (Barbour, 2001) of a portion of the polymeric chain of Sn(C7H7)3(C8H7O2) at the 70% probability level (fourth formula unit); hydrogen atoms are drawn as spheres of arbitrary radius.

## supplementary crystallographic information

### Comment

Trialkyltin carboxylates generally adopt five-coordinate, carboxylate-bridged structures (Ng *et al.*, 1988), as exemplified by tribenzyltin acetate, which is polymeric with a short covalent and a long dative Sn–O bond [2.131 (2), 2.559 (2) Å] (Ferguson *et al.*, 1995). Other trialkyltin carboxylates have less uneven bonds; however, the repeat distance of such polymers is generally about 5.19 Å. This is approximately the value found in the tribenzyltin cinnamate (Scheme I). In tribenzyltin cinnamate, the Sn^IV^ atom of the four independent carboxylate-bridged polymeric [Sn(C_7_H_7_)_3_(C_9_H_7_O_2_)]_n_, chains exists in a *trans*-C_3_SnO_2_ trigonal bipyramidal geometry [covalent Sn–O_av._ 2.167 Å, dative Sn–O_av._ 2.361 Å; O–Sn–O_av._ 169.6 °].. Each chain propagates as a helical chain along the *b*-axis of the monoclinic unit cell (Figs. 1 - 4). The repeat distance is half the *b*-axial length, *i.e.*, 5.12 Å. Two formula units are disposed about a false center-of-inversion with respect to the other two so that the space group emulates a centric space group.

Tribenzyltin cinnamate as well as tribenzyltin *p*-nitrocinnamate (Thong *et al.*, 2008) both display similar covalent and dative tin-oxygen bonds, and their repeat distances are nearly identical.

The title compoud was refined in the polar *P*2_1_ space group; *PLATON* (Spek, 2009) suggested the centric *P*2_1_/*a* space group with a 100% probability; the checking program examines the atomic coordinates. In fact, the *h*0*l* reflections that define an *a*-glide, though weak, are not systematically absent. The solution in the centric space group did not converge to a satisfactory *R*-index although the measurements are of high quality. Finally, the Flack parameter refined to 0.00 (2), an indication of the polar nature of the *P*2_1_ space group. There are examples of incorrectly assigned space groups for which the Flack parameter also refined to zero but the present diffraction dataset has a large number of Friedel pairs that attest to the correctness of Flack parameter. As an additional check, the absolute structure parameter *y* (Hooft *et al.*, 2008) was calculated using *PLATON* (Spek, 2009). The resulting value of *y* is 0.00 (1), which indicates that the absolute structure has probably been determined correctly.

The assignment of *P*2_1_ space groups is sometimes problematic. In this case, the *h*0*l* reflections are merely weak whereas in other cases, the *h*0*l* reflections are indeed systematically absent. Clemente & Marzotto (2003, 2004) have presented examples of compounds refined in the *P*2_1_ space group that should be described in higher-symmetry space groups.

### Experimental

Tribenzyltin hydroxide was first prepared by the base hydrolysis of tribenzyltin chloride with 10% sodium hydroxide solution. The hydroxide (0.40 g, 1 mmol) and cinnamic acid (0.15 g, 1 mmol) were heated in ethanol (100 ml) until the reactants dissolved completely. The solution was then filtered and colorless crystals were obtained upon slow evaporation of the solvent.

### Refinement

Carbon-bound H-atoms were placed in calculated positions (C—H 0.95 to 0.99 Å) and were included in the refinement in the riding model approximation, with *U*(H) set to 1.2 times *U*_eq_(C). The Flack parameter was calculated from 10328 Friedel pairs.

The *h*0*l* reflections that define an *a*-glide, though weak, are not systematically absent. Furthermore, the solution in the centric space group did not converge to a satisfactory *R*-index although the measurements are of high quality. Finally, the Flack parameter refined to 0.00 (2), an indication of the polar nature of the *P*2_1_ space group.

### Crystal data

**Table:**

[Sn(C_7_H_7_)_3_(C_9_H_7_O_2_)]	*F*(000) = 2192
*M**_r_* = 539.21	*D*_x_ = 1.430 Mg m^−^^3^
Monoclinic, *P*2_1_	Mo *K*α radiation, λ = 0.71073 Å
Hall symbol: P 2yb	Cell parameters from 8518 reflections
*a* = 19.5847 (3) Å	θ = 2.3–24.6°
*b* = 10.2404 (2) Å	µ = 1.04 mm^−^^1^
*c* = 26.6224 (4) Å	*T* = 100 K
β = 110.2838 (8)°	Block, colorless
*V* = 5008.15 (15) Å^3^	0.20 × 0.10 × 0.10 mm
*Z* = 8	

### Data collection

**Table:**

Bruker SMART APEX diffractometer	22492 independent reflections
Radiation source: fine-focus sealed tube	19107 reflections with *I* > 2σ(*I*)
graphite	*R*_int_ = 0.049
ω scans	θ_max_ = 27.5°, θ_min_ = 0.8°
Absorption correction: multi-scan (*SADABS*; Sheldrick, 1996)	*h* = −25→25
*T*_min_ = 0.818, *T*_max_ = 0.903	*k* = −13→13
47886 measured reflections	*l* = −34→34

### Refinement

**Table:**

Refinement on *F*^2^	Secondary atom site location: difference Fourier map
Least-squares matrix: full	Hydrogen site location: inferred from neighbouring sites
*R*[*F*^2^ > 2σ(*F*^2^)] = 0.042	H-atom parameters constrained
*wR*(*F*^2^) = 0.087	*w* = 1/[σ^2^(*F*_o_^2^) + (0.0349*P*)^2^] where *P* = (*F*_o_^2^ + 2*F*_c_^2^)/3
*S* = 0.97	(Δ/σ)_max_ = 0.001
22492 reflections	Δρ_max_ = 0.83 e Å^−^^3^
1189 parameters	Δρ_min_ = −0.79 e Å^−^^3^
1 restraint	Absolute structure: Flack (1983), 10328 Friedel pairs
Primary atom site location: structure-invariant direct methods	Flack parameter: 0.00 (2)

### Fractional atomic coordinates and isotropic or equivalent isotropic displacement parameters (Å^2^)

**Table:**

	*x*	*y*	*z*	*U*_iso_*/*U*_eq_	
Sn1	0.494183 (19)	0.50003 (3)	0.024475 (14)	0.01855 (8)	
Sn2	0.473268 (18)	0.73095 (3)	0.477818 (12)	0.01602 (7)	
Sn3	0.028304 (19)	0.67354 (3)	0.517422 (13)	0.01674 (8)	
Sn4	0.005351 (19)	0.96457 (3)	0.977122 (13)	0.01520 (8)	
O1	0.4760 (2)	0.6546 (3)	0.07388 (13)	0.0221 (8)	
O2	0.4912 (2)	0.8102 (4)	0.02052 (14)	0.0229 (8)	
O3	0.41043 (19)	0.8820 (3)	0.42395 (13)	0.0203 (8)	
O4	0.47228 (19)	1.0424 (3)	0.47598 (13)	0.0193 (8)	
O5	0.09833 (19)	0.5343 (3)	0.57185 (13)	0.0209 (8)	
O6	0.0365 (2)	0.3609 (4)	0.52990 (14)	0.0230 (8)	
O7	0.01843 (19)	0.8125 (3)	0.92420 (13)	0.0190 (8)	
O8	0.00660 (19)	0.6558 (4)	0.97772 (12)	0.0202 (8)	
C1	0.6043 (3)	0.5568 (5)	0.0369 (3)	0.0340 (15)	
H1A	0.6084	0.5800	0.0020	0.041*	
H1B	0.6162	0.6356	0.0599	0.041*	
C2	0.6588 (3)	0.4511 (5)	0.0628 (2)	0.0232 (12)	
C3	0.6710 (4)	0.4125 (8)	0.1158 (3)	0.0452 (19)	
H3	0.6469	0.4562	0.1364	0.054*	
C4	0.7182 (4)	0.3103 (9)	0.1379 (3)	0.059 (2)	
H4	0.7246	0.2824	0.1733	0.070*	
C5	0.7559 (4)	0.2487 (6)	0.1100 (3)	0.0441 (18)	
H5	0.7887	0.1798	0.1259	0.053*	
C6	0.7456 (4)	0.2872 (6)	0.0598 (3)	0.0368 (15)	
H6	0.7726	0.2465	0.0405	0.044*	
C7	0.6965 (3)	0.3852 (5)	0.0354 (2)	0.0286 (13)	
H7	0.6887	0.4074	−0.0008	0.034*	
C8	0.4075 (3)	0.5580 (5)	−0.0464 (2)	0.0246 (12)	
H8A	0.3876	0.6422	−0.0394	0.030*	
H8B	0.4276	0.5727	−0.0753	0.030*	
C9	0.3466 (3)	0.4614 (5)	−0.0655 (2)	0.0235 (12)	
C10	0.3005 (3)	0.4389 (5)	−0.0364 (3)	0.0299 (14)	
H10	0.3088	0.4844	−0.0037	0.036*	
C11	0.2438 (3)	0.3526 (6)	−0.0539 (3)	0.0399 (16)	
H11	0.2134	0.3382	−0.0333	0.048*	
C12	0.2307 (4)	0.2866 (6)	−0.1016 (3)	0.054 (2)	
H12	0.1910	0.2277	−0.1140	0.064*	
C13	0.2759 (4)	0.3064 (7)	−0.1316 (3)	0.0476 (18)	
H13	0.2676	0.2608	−0.1643	0.057*	
C14	0.3328 (4)	0.3931 (6)	−0.1128 (3)	0.0338 (15)	
H14	0.3637	0.4065	−0.1331	0.041*	
C15	0.4723 (3)	0.3541 (5)	0.0752 (2)	0.0249 (12)	
H15A	0.4236	0.3174	0.0556	0.030*	
H15B	0.5079	0.2826	0.0794	0.030*	
C16	0.4741 (3)	0.3910 (5)	0.1306 (2)	0.0205 (11)	
C17	0.4169 (3)	0.4589 (5)	0.1382 (2)	0.0234 (12)	
H17	0.3763	0.4841	0.1080	0.028*	
C18	0.4183 (3)	0.4900 (5)	0.1887 (2)	0.0259 (12)	
H18	0.3779	0.5340	0.1929	0.031*	
C19	0.4764 (4)	0.4590 (6)	0.2329 (2)	0.0342 (14)	
H19	0.4775	0.4838	0.2676	0.041*	
C20	0.5333 (4)	0.3916 (6)	0.2266 (2)	0.0355 (14)	
H20	0.5738	0.3676	0.2571	0.043*	
C21	0.5321 (3)	0.3580 (6)	0.1756 (2)	0.0311 (13)	
H21	0.5721	0.3116	0.1718	0.037*	
C22	0.4771 (3)	0.7744 (5)	0.0616 (2)	0.0207 (12)	
C23	0.4620 (3)	0.8704 (5)	0.0971 (2)	0.0230 (12)	
H23	0.4609	0.9605	0.0882	0.028*	
C24	0.4498 (3)	0.8353 (5)	0.1416 (2)	0.0196 (11)	
H24	0.4505	0.7443	0.1487	0.024*	
C25	0.4353 (3)	0.9221 (5)	0.1807 (2)	0.0185 (11)	
C26	0.4330 (3)	0.8699 (6)	0.2287 (2)	0.0265 (12)	
H26	0.4414	0.7792	0.2356	0.032*	
C27	0.4188 (3)	0.9474 (5)	0.2661 (2)	0.0274 (13)	
H27	0.4174	0.9103	0.2985	0.033*	
C28	0.4065 (3)	1.0788 (5)	0.2565 (2)	0.0248 (12)	
H28	0.3959	1.1323	0.2820	0.030*	
C29	0.4094 (3)	1.1328 (5)	0.2102 (2)	0.0293 (13)	
H29	0.4016	1.2239	0.2039	0.035*	
C30	0.4236 (3)	1.0553 (5)	0.1727 (2)	0.0269 (13)	
H30	0.4255	1.0938	0.1407	0.032*	
C31	0.4127 (3)	0.5818 (5)	0.4236 (2)	0.0247 (12)	
H31A	0.4468	0.5336	0.4104	0.030*	
H31B	0.3942	0.5193	0.4442	0.030*	
C32	0.3498 (3)	0.6254 (4)	0.3762 (2)	0.0201 (11)	
C33	0.3584 (3)	0.6618 (6)	0.32802 (19)	0.0274 (12)	
H33	0.4051	0.6545	0.3249	0.033*	
C34	0.3016 (4)	0.7078 (6)	0.2854 (2)	0.0386 (16)	
H34	0.3096	0.7337	0.2536	0.046*	
C35	0.2325 (3)	0.7166 (5)	0.2882 (2)	0.0320 (14)	
H35	0.1930	0.7481	0.2586	0.038*	
C36	0.2216 (3)	0.6789 (6)	0.3348 (2)	0.0295 (12)	
H36	0.1744	0.6831	0.3373	0.035*	
C37	0.2801 (3)	0.6352 (5)	0.3778 (2)	0.0227 (12)	
H37	0.2721	0.6109	0.4098	0.027*	
C38	0.4420 (3)	0.7885 (5)	0.5446 (2)	0.0228 (11)	
H38A	0.4860	0.8185	0.5740	0.027*	
H38B	0.4082	0.8635	0.5336	0.027*	
C39	0.4064 (3)	0.6834 (5)	0.56550 (19)	0.0190 (11)	
C40	0.3371 (3)	0.6403 (5)	0.5355 (2)	0.0233 (12)	
H40	0.3121	0.6802	0.5019	0.028*	
C41	0.3034 (3)	0.5407 (5)	0.5532 (2)	0.0289 (13)	
H41	0.2563	0.5116	0.5314	0.035*	
C42	0.3376 (3)	0.4838 (5)	0.6021 (2)	0.0292 (13)	
H42	0.3145	0.4157	0.6144	0.035*	
C43	0.4055 (3)	0.5260 (5)	0.6331 (2)	0.0288 (13)	
H43	0.4294	0.4875	0.6671	0.035*	
C44	0.4395 (3)	0.6249 (5)	0.6149 (2)	0.0235 (12)	
H44	0.4866	0.6531	0.6369	0.028*	
C45	0.5767 (3)	0.7892 (5)	0.4745 (2)	0.0188 (11)	
H45A	0.5720	0.8779	0.4588	0.023*	
H45B	0.6123	0.7939	0.5114	0.023*	
C46	0.6058 (3)	0.6986 (4)	0.4421 (2)	0.0200 (11)	
C47	0.5753 (3)	0.6973 (5)	0.3862 (2)	0.0274 (13)	
H47	0.5345	0.7513	0.3688	0.033*	
C48	0.6036 (4)	0.6189 (6)	0.3561 (2)	0.0339 (15)	
H48	0.5815	0.6187	0.3182	0.041*	
C49	0.6631 (4)	0.5412 (6)	0.3802 (2)	0.0359 (15)	
H49	0.6832	0.4894	0.3591	0.043*	
C50	0.6932 (3)	0.5396 (5)	0.4352 (2)	0.0323 (14)	
H50	0.7336	0.4844	0.4523	0.039*	
C51	0.6652 (3)	0.6174 (5)	0.4658 (2)	0.0255 (12)	
H51	0.6869	0.6154	0.5037	0.031*	
C52	0.4226 (3)	1.0026 (5)	0.43487 (18)	0.0173 (10)	
C53	0.3748 (3)	1.0960 (5)	0.3960 (2)	0.0209 (12)	
H53	0.3868	1.1863	0.4000	0.025*	
C54	0.3165 (3)	1.0594 (5)	0.35629 (19)	0.0186 (11)	
H54	0.3071	0.9681	0.3537	0.022*	
C55	0.2638 (3)	1.1401 (5)	0.3153 (2)	0.0197 (11)	
C56	0.2761 (3)	1.2723 (5)	0.3087 (2)	0.0246 (13)	
H56	0.3199	1.3127	0.3307	0.030*	
C57	0.2234 (4)	1.3452 (6)	0.2693 (2)	0.0369 (16)	
H57	0.2316	1.4351	0.2647	0.044*	
C58	0.1606 (4)	1.2879 (7)	0.2376 (2)	0.0394 (17)	
H58	0.1255	1.3381	0.2109	0.047*	
C59	0.1474 (3)	1.1582 (7)	0.2438 (2)	0.0391 (16)	
H59	0.1030	1.1191	0.2221	0.047*	
C60	0.1993 (3)	1.0849 (6)	0.2821 (2)	0.0286 (13)	
H60	0.1905	0.9947	0.2857	0.034*	
C61	0.0888 (3)	0.8352 (5)	0.5635 (2)	0.0232 (12)	
H61A	0.1113	0.8824	0.5407	0.028*	
H61B	0.0534	0.8956	0.5701	0.028*	
C62	0.1474 (3)	0.8092 (5)	0.6162 (2)	0.0187 (11)	
C63	0.2155 (3)	0.7625 (5)	0.6187 (2)	0.0227 (12)	
H63	0.2243	0.7460	0.5864	0.027*	
C64	0.2702 (3)	0.7397 (5)	0.6669 (2)	0.0258 (12)	
H64	0.3163	0.7088	0.6676	0.031*	
C65	0.2581 (3)	0.7618 (5)	0.7140 (2)	0.0292 (14)	
H65	0.2951	0.7443	0.7474	0.035*	
C66	0.1910 (3)	0.8099 (6)	0.7120 (2)	0.0357 (14)	
H66	0.1827	0.8280	0.7444	0.043*	
C67	0.1363 (3)	0.8318 (5)	0.6637 (2)	0.0251 (12)	
H67	0.0904	0.8629	0.6632	0.030*	
C68	−0.0718 (3)	0.6155 (5)	0.5268 (2)	0.0214 (11)	
H68A	−0.1100	0.6092	0.4909	0.026*	
H68B	−0.0654	0.5275	0.5432	0.026*	
C69	−0.0970 (3)	0.7083 (4)	0.5609 (2)	0.0213 (12)	
C70	−0.0571 (3)	0.7209 (6)	0.6152 (2)	0.0266 (12)	
H70	−0.0123	0.6754	0.6300	0.032*	
C71	−0.0816 (3)	0.7986 (6)	0.6478 (2)	0.0310 (14)	
H71	−0.0540	0.8048	0.6849	0.037*	
C72	−0.1456 (3)	0.8671 (6)	0.6268 (2)	0.0327 (14)	
H72	−0.1620	0.9210	0.6493	0.039*	
C73	−0.1860 (3)	0.8574 (6)	0.5729 (2)	0.0355 (15)	
H73	−0.2303	0.9045	0.5583	0.043*	
C74	−0.1613 (3)	0.7780 (5)	0.5399 (2)	0.0259 (13)	
H74	−0.1889	0.7719	0.5028	0.031*	
C75	0.0512 (3)	0.6192 (5)	0.44671 (19)	0.0213 (11)	
H75A	0.0834	0.5418	0.4549	0.026*	
H75B	0.0051	0.5944	0.4182	0.026*	
C76	0.0868 (3)	0.7266 (5)	0.42624 (19)	0.0206 (11)	
C77	0.0490 (3)	0.7940 (5)	0.3790 (2)	0.0220 (12)	
H77	0.0005	0.7695	0.3588	0.026*	
C78	0.0817 (3)	0.8971 (5)	0.3613 (2)	0.0283 (13)	
H78	0.0557	0.9412	0.3289	0.034*	
C79	0.1511 (3)	0.9339 (5)	0.3908 (2)	0.0254 (13)	
H79	0.1724	1.0063	0.3794	0.030*	
C80	0.1908 (3)	0.8678 (5)	0.4368 (2)	0.0266 (12)	
H80	0.2397	0.8921	0.4564	0.032*	
C81	0.1583 (3)	0.7657 (5)	0.4541 (2)	0.0269 (13)	
H81	0.1855	0.7206	0.4859	0.032*	
C82	0.0870 (3)	0.4108 (5)	0.5670 (2)	0.0192 (11)	
C83	0.1389 (3)	0.3275 (5)	0.6085 (2)	0.0197 (11)	
H83	0.1327	0.2354	0.6062	0.024*	
C84	0.1945 (3)	0.3780 (5)	0.6491 (2)	0.0215 (11)	
H84	0.1982	0.4705	0.6497	0.026*	
C85	0.2497 (3)	0.3101 (5)	0.6923 (2)	0.0215 (11)	
C86	0.3104 (3)	0.3805 (5)	0.7234 (2)	0.0222 (11)	
H86	0.3130	0.4712	0.7168	0.027*	
C87	0.3672 (3)	0.3224 (5)	0.7637 (2)	0.0266 (13)	
H87	0.4087	0.3719	0.7837	0.032*	
C88	0.3628 (3)	0.1911 (6)	0.7745 (2)	0.0299 (14)	
H88	0.4014	0.1504	0.8021	0.036*	
C89	0.3017 (3)	0.1181 (6)	0.7448 (2)	0.0309 (13)	
H89	0.2984	0.0286	0.7530	0.037*	
C90	0.2466 (3)	0.1755 (6)	0.70375 (19)	0.0233 (11)	
H90	0.2061	0.1247	0.6829	0.028*	
C91	−0.1039 (3)	0.9099 (5)	0.9703 (2)	0.0185 (11)	
H91A	−0.1149	0.8220	0.9540	0.022*	
H91B	−0.1074	0.9056	1.0065	0.022*	
C92	−0.1584 (3)	1.0044 (5)	0.9372 (2)	0.0211 (11)	
C93	−0.1720 (3)	1.0147 (6)	0.8833 (2)	0.0308 (14)	
H93	−0.1473	0.9579	0.8671	0.037*	
C94	−0.2202 (4)	1.1044 (7)	0.8519 (2)	0.0369 (16)	
H94	−0.2286	1.1085	0.8146	0.044*	
C95	−0.2562 (3)	1.1883 (6)	0.8745 (2)	0.0325 (14)	
H95	−0.2886	1.2519	0.8530	0.039*	
C96	−0.2451 (3)	1.1797 (6)	0.9285 (2)	0.0275 (12)	
H96	−0.2706	1.2359	0.9443	0.033*	
C97	−0.1961 (3)	1.0882 (5)	0.9599 (2)	0.0256 (12)	
H97	−0.1882	1.0826	0.9971	0.031*	
C98	0.0961 (3)	0.9066 (5)	1.0462 (2)	0.0240 (12)	
H98A	0.0783	0.8867	1.0759	0.029*	
H98B	0.1174	0.8255	1.0376	0.029*	
C99	0.1547 (3)	1.0089 (5)	1.0646 (2)	0.0205 (11)	
C100	0.1679 (3)	1.0771 (6)	1.1124 (2)	0.0279 (13)	
H100	0.1397	1.0594	1.1344	0.034*	
C101	0.2226 (3)	1.1710 (6)	1.1278 (2)	0.0376 (15)	
H101	0.2324	1.2151	1.1610	0.045*	
C102	0.2626 (3)	1.2015 (6)	1.0962 (3)	0.0395 (16)	
H102	0.2981	1.2688	1.1064	0.047*	
C103	0.2507 (3)	1.1338 (6)	1.0499 (2)	0.0364 (15)	
H103	0.2796	1.1519	1.0285	0.044*	
C104	0.1969 (3)	1.0384 (6)	1.0336 (2)	0.0297 (13)	
H104	0.1889	0.9931	1.0009	0.036*	
C105	0.0206 (3)	1.1114 (5)	0.92432 (19)	0.0197 (11)	
H10A	−0.0204	1.1736	0.9164	0.024*	
H10B	0.0655	1.1603	0.9442	0.024*	
C106	0.0264 (3)	1.0695 (5)	0.8717 (2)	0.0184 (11)	
C107	0.0906 (3)	1.0169 (5)	0.8689 (2)	0.0214 (11)	
H107	0.1314	1.0073	0.9009	0.026*	
C108	0.0966 (3)	0.9781 (5)	0.8207 (2)	0.0290 (13)	
H108	0.1411	0.9438	0.8196	0.035*	
C109	0.0365 (4)	0.9903 (6)	0.7740 (2)	0.0333 (14)	
H109	0.0397	0.9631	0.7408	0.040*	
C110	−0.0274 (4)	1.0413 (6)	0.7757 (2)	0.0343 (14)	
H110	−0.0684	1.0492	0.7437	0.041*	
C111	−0.0321 (3)	1.0813 (5)	0.8239 (2)	0.0256 (12)	
H111	−0.0764	1.1178	0.8245	0.031*	
C112	0.0173 (3)	0.6923 (5)	0.9362 (2)	0.0186 (11)	
C113	0.0302 (3)	0.5973 (5)	0.89888 (19)	0.0207 (11)	
H113	0.0291	0.5068	0.9063	0.025*	
C114	0.0434 (3)	0.6336 (5)	0.85527 (18)	0.0171 (10)	
H114	0.0420	0.7248	0.8484	0.021*	
C115	0.0600 (3)	0.5495 (5)	0.8165 (2)	0.0209 (11)	
C116	0.0818 (3)	0.6041 (6)	0.7767 (2)	0.0288 (13)	
H116	0.0833	0.6964	0.7737	0.035*	
C117	0.1014 (3)	0.5260 (6)	0.7412 (2)	0.0336 (14)	
H117	0.1169	0.5651	0.7146	0.040*	
C118	0.0984 (3)	0.3923 (6)	0.7444 (2)	0.0283 (13)	
H118	0.1114	0.3391	0.7200	0.034*	
C119	0.0764 (4)	0.3354 (5)	0.7832 (2)	0.0370 (16)	
H119	0.0744	0.2430	0.7856	0.044*	
C120	0.0571 (4)	0.4140 (5)	0.8189 (2)	0.0334 (15)	
H120	0.0416	0.3743	0.8454	0.040*	

### Atomic displacement parameters (Å^2^)

**Table:**

	*U*^11^	*U*^22^	*U*^33^	*U*^12^	*U*^13^	*U*^23^
Sn1	0.02054 (19)	0.01135 (17)	0.02684 (18)	−0.00035 (15)	0.01212 (16)	−0.00068 (14)
Sn2	0.01615 (18)	0.01263 (15)	0.01848 (17)	−0.00024 (14)	0.00497 (14)	0.00100 (14)
Sn3	0.01770 (18)	0.01492 (16)	0.01692 (16)	−0.00074 (15)	0.00515 (14)	0.00023 (14)
Sn4	0.01741 (19)	0.01094 (17)	0.01841 (17)	0.00012 (13)	0.00769 (15)	−0.00003 (13)
O1	0.033 (2)	0.0103 (19)	0.0251 (19)	−0.0041 (16)	0.0131 (17)	−0.0019 (15)
O2	0.030 (2)	0.012 (2)	0.032 (2)	0.0001 (16)	0.0175 (18)	0.0019 (15)
O3	0.022 (2)	0.0082 (17)	0.0250 (19)	0.0011 (14)	0.0010 (16)	−0.0017 (14)
O4	0.021 (2)	0.0164 (18)	0.0198 (18)	−0.0020 (15)	0.0057 (16)	−0.0016 (14)
O5	0.025 (2)	0.0141 (19)	0.0193 (18)	−0.0006 (15)	0.0021 (16)	−0.0014 (14)
O6	0.023 (2)	0.0195 (19)	0.0224 (19)	−0.0011 (15)	0.0025 (16)	−0.0008 (15)
O7	0.029 (2)	0.0096 (18)	0.0227 (18)	0.0011 (15)	0.0146 (16)	0.0020 (14)
O8	0.026 (2)	0.017 (2)	0.0214 (18)	−0.0013 (16)	0.0129 (17)	0.0032 (15)
C1	0.026 (3)	0.016 (3)	0.063 (4)	−0.003 (2)	0.019 (3)	−0.006 (3)
C2	0.019 (3)	0.021 (3)	0.029 (3)	−0.011 (2)	0.008 (3)	−0.005 (2)
C3	0.024 (4)	0.084 (6)	0.029 (4)	−0.008 (3)	0.011 (3)	−0.017 (3)
C4	0.033 (4)	0.113 (7)	0.022 (3)	−0.014 (4)	0.000 (3)	0.021 (4)
C5	0.026 (4)	0.038 (4)	0.053 (4)	−0.009 (3)	−0.006 (3)	0.014 (3)
C6	0.038 (4)	0.029 (3)	0.038 (4)	0.000 (3)	0.006 (3)	−0.008 (3)
C7	0.034 (4)	0.023 (3)	0.033 (3)	0.003 (3)	0.017 (3)	0.000 (2)
C8	0.025 (3)	0.022 (3)	0.028 (3)	0.003 (2)	0.010 (3)	0.002 (2)
C9	0.029 (3)	0.015 (3)	0.028 (3)	0.003 (2)	0.012 (3)	0.003 (2)
C10	0.026 (3)	0.032 (3)	0.036 (3)	0.004 (2)	0.015 (3)	0.005 (2)
C11	0.029 (4)	0.040 (4)	0.053 (4)	0.002 (3)	0.016 (3)	0.016 (3)
C12	0.024 (4)	0.024 (3)	0.099 (6)	−0.009 (3)	0.004 (4)	0.001 (4)
C13	0.036 (4)	0.038 (4)	0.061 (5)	−0.004 (3)	0.008 (4)	−0.022 (4)
C14	0.028 (4)	0.040 (4)	0.037 (4)	−0.004 (3)	0.015 (3)	−0.009 (3)
C15	0.031 (3)	0.013 (3)	0.031 (3)	0.003 (2)	0.012 (3)	0.002 (2)
C16	0.024 (3)	0.015 (3)	0.024 (3)	−0.003 (2)	0.011 (2)	0.004 (2)
C17	0.025 (3)	0.013 (2)	0.030 (3)	−0.003 (2)	0.008 (2)	0.002 (2)
C18	0.028 (3)	0.017 (3)	0.037 (3)	−0.005 (2)	0.017 (3)	−0.002 (2)
C19	0.047 (4)	0.031 (3)	0.029 (3)	−0.016 (3)	0.019 (3)	−0.007 (3)
C20	0.032 (4)	0.046 (4)	0.026 (3)	−0.001 (3)	0.006 (3)	0.001 (3)
C21	0.028 (3)	0.031 (3)	0.037 (3)	0.005 (2)	0.014 (3)	0.004 (3)
C22	0.019 (3)	0.019 (3)	0.025 (3)	−0.003 (2)	0.008 (2)	−0.002 (2)
C23	0.024 (3)	0.011 (3)	0.039 (3)	−0.004 (2)	0.018 (3)	−0.006 (2)
C24	0.019 (3)	0.011 (2)	0.029 (3)	−0.001 (2)	0.007 (2)	0.000 (2)
C25	0.017 (3)	0.014 (3)	0.025 (3)	0.000 (2)	0.007 (2)	−0.001 (2)
C26	0.024 (3)	0.024 (3)	0.034 (3)	−0.002 (2)	0.013 (2)	0.002 (2)
C27	0.031 (3)	0.030 (3)	0.026 (3)	−0.005 (2)	0.016 (3)	0.000 (2)
C28	0.026 (3)	0.025 (3)	0.025 (3)	−0.002 (2)	0.011 (2)	−0.011 (2)
C29	0.040 (4)	0.019 (3)	0.029 (3)	0.002 (2)	0.011 (3)	−0.003 (2)
C30	0.042 (4)	0.021 (3)	0.021 (3)	0.005 (2)	0.015 (3)	0.003 (2)
C31	0.023 (3)	0.018 (3)	0.027 (3)	−0.004 (2)	0.002 (2)	0.000 (2)
C32	0.018 (3)	0.011 (2)	0.028 (3)	−0.0044 (19)	0.003 (2)	−0.005 (2)
C33	0.026 (3)	0.035 (3)	0.022 (3)	−0.007 (3)	0.009 (2)	−0.009 (3)
C34	0.050 (4)	0.043 (4)	0.017 (3)	−0.022 (3)	0.003 (3)	−0.002 (2)
C35	0.036 (4)	0.019 (3)	0.027 (3)	−0.009 (3)	−0.008 (3)	−0.002 (2)
C36	0.023 (3)	0.032 (3)	0.027 (3)	0.005 (3)	0.002 (2)	−0.010 (3)
C37	0.021 (3)	0.026 (3)	0.020 (3)	−0.005 (2)	0.006 (2)	0.000 (2)
C38	0.023 (3)	0.019 (3)	0.025 (3)	−0.005 (2)	0.006 (2)	0.002 (2)
C39	0.019 (3)	0.019 (2)	0.022 (2)	0.004 (2)	0.011 (2)	0.001 (2)
C40	0.017 (3)	0.028 (3)	0.025 (3)	0.006 (2)	0.007 (2)	0.003 (2)
C41	0.018 (3)	0.034 (3)	0.039 (3)	−0.003 (2)	0.015 (3)	−0.003 (3)
C42	0.030 (3)	0.024 (3)	0.039 (3)	0.002 (2)	0.019 (3)	0.003 (2)
C43	0.036 (3)	0.029 (3)	0.028 (3)	0.005 (3)	0.020 (3)	0.008 (2)
C44	0.016 (3)	0.029 (3)	0.026 (3)	0.000 (2)	0.008 (2)	−0.001 (2)
C45	0.015 (3)	0.018 (2)	0.023 (3)	−0.001 (2)	0.007 (2)	0.002 (2)
C46	0.025 (3)	0.010 (3)	0.025 (3)	−0.0059 (19)	0.009 (2)	−0.0044 (18)
C47	0.029 (3)	0.023 (3)	0.030 (3)	−0.002 (2)	0.010 (3)	−0.003 (2)
C48	0.039 (4)	0.035 (3)	0.028 (3)	−0.010 (3)	0.014 (3)	−0.008 (3)
C49	0.047 (4)	0.026 (3)	0.045 (4)	0.006 (3)	0.029 (3)	−0.004 (3)
C50	0.035 (3)	0.025 (3)	0.044 (3)	0.010 (3)	0.022 (3)	0.009 (3)
C51	0.028 (3)	0.024 (3)	0.028 (3)	0.004 (2)	0.015 (3)	0.007 (2)
C52	0.018 (3)	0.019 (2)	0.017 (2)	0.002 (2)	0.008 (2)	0.002 (2)
C53	0.030 (3)	0.013 (2)	0.025 (3)	0.004 (2)	0.016 (3)	−0.002 (2)
C54	0.025 (3)	0.014 (2)	0.017 (3)	−0.001 (2)	0.007 (2)	0.0006 (19)
C55	0.022 (3)	0.019 (3)	0.020 (3)	0.005 (2)	0.010 (2)	0.003 (2)
C56	0.032 (3)	0.018 (3)	0.026 (3)	0.009 (2)	0.014 (3)	0.004 (2)
C57	0.064 (5)	0.019 (3)	0.035 (3)	0.020 (3)	0.027 (3)	0.009 (3)
C58	0.042 (4)	0.050 (4)	0.034 (3)	0.030 (3)	0.022 (3)	0.020 (3)
C59	0.030 (3)	0.062 (5)	0.023 (3)	0.013 (3)	0.007 (3)	0.002 (3)
C60	0.035 (4)	0.031 (3)	0.021 (3)	0.002 (3)	0.010 (3)	0.003 (2)
C61	0.022 (3)	0.017 (3)	0.025 (3)	−0.001 (2)	0.000 (2)	0.003 (2)
C62	0.027 (3)	0.011 (2)	0.017 (3)	−0.008 (2)	0.007 (2)	−0.0025 (19)
C63	0.023 (3)	0.027 (3)	0.020 (3)	−0.001 (2)	0.009 (2)	−0.001 (2)
C64	0.022 (3)	0.019 (2)	0.033 (3)	0.000 (2)	0.005 (2)	−0.004 (2)
C65	0.024 (3)	0.033 (4)	0.020 (3)	−0.004 (2)	−0.006 (2)	0.002 (2)
C66	0.031 (3)	0.057 (4)	0.022 (3)	−0.006 (3)	0.013 (3)	−0.008 (3)
C67	0.015 (3)	0.035 (3)	0.023 (3)	0.001 (2)	0.003 (2)	−0.002 (2)
C68	0.026 (3)	0.017 (3)	0.022 (3)	0.000 (2)	0.010 (2)	−0.002 (2)
C69	0.022 (3)	0.016 (3)	0.029 (3)	−0.0063 (19)	0.014 (2)	−0.001 (2)
C70	0.026 (3)	0.027 (3)	0.025 (3)	−0.006 (2)	0.008 (2)	0.000 (2)
C71	0.035 (4)	0.035 (3)	0.024 (3)	−0.010 (3)	0.013 (3)	−0.005 (3)
C72	0.040 (4)	0.029 (3)	0.038 (3)	−0.008 (3)	0.026 (3)	−0.011 (3)
C73	0.032 (4)	0.035 (3)	0.047 (4)	0.009 (3)	0.024 (3)	0.000 (3)
C74	0.030 (4)	0.026 (3)	0.022 (3)	−0.004 (2)	0.010 (3)	−0.006 (2)
C75	0.024 (3)	0.021 (3)	0.021 (3)	0.001 (2)	0.010 (2)	0.000 (2)
C76	0.026 (3)	0.018 (2)	0.019 (2)	0.003 (2)	0.009 (2)	−0.006 (2)
C77	0.022 (3)	0.028 (3)	0.018 (3)	0.003 (2)	0.009 (2)	0.002 (2)
C78	0.035 (3)	0.022 (3)	0.031 (3)	0.010 (2)	0.015 (3)	0.007 (2)
C79	0.040 (4)	0.016 (3)	0.029 (3)	−0.003 (2)	0.023 (3)	−0.001 (2)
C80	0.027 (3)	0.028 (3)	0.029 (3)	−0.002 (2)	0.015 (2)	−0.002 (2)
C81	0.033 (4)	0.026 (3)	0.026 (3)	0.009 (2)	0.016 (3)	0.005 (2)
C82	0.018 (3)	0.024 (3)	0.016 (3)	0.000 (2)	0.007 (2)	−0.002 (2)
C83	0.017 (3)	0.018 (3)	0.023 (3)	−0.001 (2)	0.006 (2)	−0.001 (2)
C84	0.025 (3)	0.015 (3)	0.028 (3)	0.002 (2)	0.013 (2)	0.003 (2)
C85	0.019 (3)	0.026 (3)	0.022 (3)	0.004 (2)	0.009 (2)	0.002 (2)
C86	0.017 (3)	0.022 (3)	0.025 (3)	−0.002 (2)	0.006 (2)	0.003 (2)
C87	0.020 (3)	0.034 (3)	0.023 (3)	−0.002 (2)	0.005 (2)	0.001 (2)
C88	0.028 (3)	0.035 (4)	0.026 (3)	0.012 (2)	0.009 (2)	0.006 (2)
C89	0.036 (4)	0.027 (3)	0.032 (3)	0.006 (3)	0.015 (3)	0.012 (2)
C90	0.021 (3)	0.025 (3)	0.022 (3)	−0.002 (2)	0.005 (2)	0.000 (2)
C91	0.013 (3)	0.017 (3)	0.025 (3)	−0.003 (2)	0.006 (2)	0.000 (2)
C92	0.014 (3)	0.019 (3)	0.029 (3)	−0.002 (2)	0.006 (2)	−0.002 (2)
C93	0.025 (3)	0.042 (4)	0.023 (3)	0.005 (3)	0.007 (3)	0.001 (3)
C94	0.030 (4)	0.054 (4)	0.024 (3)	0.012 (3)	0.007 (3)	0.012 (3)
C95	0.025 (3)	0.028 (3)	0.037 (3)	0.005 (2)	0.001 (3)	0.006 (2)
C96	0.020 (3)	0.020 (3)	0.043 (3)	0.002 (2)	0.011 (3)	−0.005 (3)
C97	0.021 (3)	0.030 (3)	0.025 (3)	0.003 (2)	0.007 (2)	0.002 (2)
C98	0.030 (3)	0.014 (2)	0.024 (3)	−0.001 (2)	0.005 (3)	0.001 (2)
C99	0.014 (3)	0.018 (3)	0.026 (3)	0.006 (2)	0.002 (2)	0.001 (2)
C100	0.022 (3)	0.032 (3)	0.025 (3)	0.003 (2)	0.002 (3)	−0.005 (2)
C101	0.030 (3)	0.036 (3)	0.038 (3)	0.000 (3)	0.000 (3)	−0.017 (3)
C102	0.025 (3)	0.028 (4)	0.061 (4)	−0.002 (2)	0.009 (3)	−0.003 (3)
C103	0.018 (3)	0.045 (4)	0.045 (4)	−0.002 (3)	0.009 (3)	0.015 (3)
C104	0.029 (3)	0.034 (3)	0.023 (3)	0.007 (3)	0.006 (3)	−0.002 (2)
C105	0.023 (3)	0.019 (3)	0.021 (3)	0.000 (2)	0.013 (2)	−0.001 (2)
C106	0.022 (3)	0.012 (2)	0.022 (3)	−0.004 (2)	0.009 (2)	−0.0031 (19)
C107	0.029 (3)	0.013 (3)	0.022 (3)	−0.001 (2)	0.008 (2)	0.002 (2)
C108	0.044 (4)	0.014 (3)	0.036 (3)	0.002 (2)	0.023 (3)	0.004 (2)
C109	0.051 (4)	0.028 (3)	0.032 (3)	−0.016 (3)	0.027 (3)	−0.014 (3)
C110	0.039 (4)	0.038 (3)	0.022 (3)	−0.013 (3)	0.005 (3)	0.002 (2)
C111	0.027 (3)	0.028 (3)	0.023 (3)	−0.002 (2)	0.010 (3)	0.002 (2)
C112	0.017 (3)	0.012 (3)	0.026 (3)	−0.0011 (19)	0.008 (2)	−0.0026 (19)
C113	0.028 (3)	0.012 (2)	0.021 (3)	−0.001 (2)	0.007 (2)	−0.002 (2)
C114	0.018 (3)	0.010 (2)	0.020 (2)	0.0013 (19)	0.003 (2)	−0.0011 (18)
C115	0.018 (3)	0.023 (3)	0.020 (3)	0.002 (2)	0.006 (2)	−0.001 (2)
C116	0.041 (4)	0.023 (3)	0.027 (3)	−0.004 (3)	0.017 (3)	0.000 (2)
C117	0.050 (4)	0.030 (3)	0.031 (3)	0.001 (3)	0.026 (3)	0.001 (2)
C118	0.029 (3)	0.030 (3)	0.026 (3)	0.000 (3)	0.010 (3)	−0.009 (2)
C119	0.063 (5)	0.016 (3)	0.040 (4)	−0.005 (3)	0.028 (3)	−0.003 (2)
C120	0.056 (4)	0.022 (3)	0.032 (3)	−0.003 (3)	0.028 (3)	−0.002 (2)

### Geometric parameters (Å, °)

**Table:**

Sn1—C8	2.139 (5)	C52—C53	1.480 (7)
Sn1—C1	2.145 (6)	C53—C54	1.314 (7)
Sn1—C15	2.154 (5)	C53—H53	0.9500
Sn1—O1	2.165 (3)	C54—C55	1.469 (7)
Sn1—O2^i^	2.352 (4)	C54—H54	0.9500
Sn2—C45	2.142 (5)	C55—C60	1.387 (8)
Sn2—C38	2.153 (5)	C55—C56	1.397 (7)
Sn2—C31	2.153 (5)	C56—C57	1.404 (8)
Sn2—O3	2.177 (3)	C56—H56	0.9500
Sn2—O4^ii^	2.338 (3)	C57—C58	1.359 (9)
Sn3—C68	2.143 (5)	C57—H57	0.9500
Sn3—O5	2.153 (3)	C58—C59	1.375 (9)
Sn3—C75	2.154 (5)	C58—H58	0.9500
Sn3—C61	2.154 (5)	C59—C60	1.384 (8)
Sn3—O6^iii^	2.403 (4)	C59—H59	0.9500
Sn4—C105	2.149 (5)	C60—H60	0.9500
Sn4—C98	2.151 (6)	C61—C62	1.498 (7)
Sn4—C91	2.159 (5)	C61—H61A	0.9900
Sn4—O7	2.174 (3)	C61—H61B	0.9900
Sn4—O8^iv^	2.352 (4)	C62—C67	1.375 (7)
O1—C22	1.271 (6)	C62—C63	1.396 (7)
O2—C22	1.273 (6)	C63—C64	1.376 (7)
O2—Sn1^v^	2.352 (4)	C63—H63	0.9500
O3—C52	1.272 (6)	C64—C65	1.373 (8)
O4—C52	1.254 (6)	C64—H64	0.9500
O4—Sn2^vi^	2.338 (3)	C65—C66	1.388 (8)
O5—C82	1.283 (6)	C65—H65	0.9500
O6—C82	1.240 (6)	C66—C67	1.376 (7)
O6—Sn3^vii^	2.403 (4)	C66—H66	0.9500
O7—C112	1.274 (6)	C67—H67	0.9500
O8—C112	1.250 (6)	C68—C69	1.512 (7)
O8—Sn4^viii^	2.352 (4)	C68—H68A	0.9900
C1—C2	1.509 (8)	C68—H68B	0.9900
C1—H1A	0.9900	C69—C74	1.386 (8)
C1—H1B	0.9900	C69—C70	1.390 (7)
C2—C7	1.381 (8)	C70—C71	1.380 (8)
C2—C3	1.404 (9)	C70—H70	0.9500
C3—C4	1.385 (11)	C71—C72	1.375 (8)
C3—H3	0.9500	C71—H71	0.9500
C4—C5	1.369 (10)	C72—C73	1.380 (8)
C4—H4	0.9500	C72—H72	0.9500
C5—C6	1.340 (9)	C73—C74	1.400 (7)
C5—H5	0.9500	C73—H73	0.9500
C6—C7	1.386 (8)	C74—H74	0.9500
C6—H6	0.9500	C75—C76	1.502 (7)
C7—H7	0.9500	C75—H75A	0.9900
C8—C9	1.497 (8)	C75—H75B	0.9900
C8—H8A	0.9900	C76—C81	1.396 (8)
C8—H8B	0.9900	C76—C77	1.400 (7)
C9—C14	1.383 (8)	C77—C78	1.399 (7)
C9—C10	1.398 (8)	C77—H77	0.9500
C10—C11	1.369 (9)	C78—C79	1.367 (8)
C10—H10	0.9500	C78—H78	0.9500
C11—C12	1.382 (10)	C79—C80	1.380 (8)
C11—H11	0.9500	C79—H79	0.9500
C12—C13	1.398 (10)	C80—C81	1.383 (7)
C12—H12	0.9500	C80—H80	0.9500
C13—C14	1.375 (9)	C81—H81	0.9500
C13—H13	0.9500	C82—C83	1.483 (7)
C14—H14	0.9500	C83—C84	1.344 (7)
C15—C16	1.511 (7)	C83—H83	0.9500
C15—H15A	0.9900	C84—C85	1.454 (7)
C15—H15B	0.9900	C84—H84	0.9500
C16—C21	1.378 (8)	C85—C86	1.392 (7)
C16—C17	1.391 (7)	C85—C90	1.417 (7)
C17—C18	1.374 (7)	C86—C87	1.383 (7)
C17—H17	0.9500	C86—H86	0.9500
C18—C19	1.360 (8)	C87—C88	1.383 (8)
C18—H18	0.9500	C87—H87	0.9500
C19—C20	1.371 (8)	C88—C89	1.399 (8)
C19—H19	0.9500	C88—H88	0.9500
C20—C21	1.393 (8)	C89—C90	1.374 (7)
C20—H20	0.9500	C89—H89	0.9500
C21—H21	0.9500	C90—H90	0.9500
C22—C23	1.462 (7)	C91—C92	1.483 (7)
C23—C24	1.338 (7)	C91—H91A	0.9900
C23—H23	0.9500	C91—H91B	0.9900
C24—C25	1.468 (7)	C92—C93	1.369 (7)
C24—H24	0.9500	C92—C97	1.399 (7)
C25—C30	1.388 (7)	C93—C94	1.374 (8)
C25—C26	1.401 (7)	C93—H93	0.9500
C26—C27	1.376 (7)	C94—C95	1.376 (8)
C26—H26	0.9500	C94—H94	0.9500
C27—C28	1.375 (7)	C95—C96	1.382 (7)
C27—H27	0.9500	C95—H95	0.9500
C28—C29	1.371 (7)	C96—C97	1.392 (8)
C28—H28	0.9500	C96—H96	0.9500
C29—C30	1.376 (7)	C97—H97	0.9500
C29—H29	0.9500	C98—C99	1.505 (8)
C30—H30	0.9500	C98—H98A	0.9900
C31—C32	1.495 (7)	C98—H98B	0.9900
C31—H31A	0.9900	C99—C104	1.388 (8)
C31—H31B	0.9900	C99—C100	1.396 (7)
C32—C37	1.385 (7)	C100—C101	1.391 (9)
C32—C33	1.400 (7)	C100—H100	0.9500
C33—C34	1.369 (8)	C101—C102	1.371 (9)
C33—H33	0.9500	C101—H101	0.9500
C34—C35	1.385 (9)	C102—C103	1.361 (9)
C34—H34	0.9500	C102—H102	0.9500
C35—C36	1.384 (8)	C103—C104	1.391 (8)
C35—H35	0.9500	C103—H103	0.9500
C36—C37	1.383 (7)	C104—H104	0.9500
C36—H36	0.9500	C105—C106	1.506 (7)
C37—H37	0.9500	C105—H10A	0.9900
C38—C39	1.491 (7)	C105—H10B	0.9900
C38—H38A	0.9900	C106—C111	1.392 (7)
C38—H38B	0.9900	C106—C107	1.393 (7)
C39—C44	1.385 (7)	C107—C108	1.387 (7)
C39—C40	1.387 (7)	C107—H107	0.9500
C40—C41	1.385 (7)	C108—C109	1.388 (8)
C40—H40	0.9500	C108—H108	0.9500
C41—C42	1.371 (8)	C109—C110	1.372 (9)
C41—H41	0.9500	C109—H109	0.9500
C42—C43	1.368 (8)	C110—C111	1.380 (7)
C42—H42	0.9500	C110—H110	0.9500
C43—C44	1.388 (7)	C111—H111	0.9500
C43—H43	0.9500	C112—C113	1.475 (7)
C44—H44	0.9500	C113—C114	1.327 (7)
C45—C46	1.507 (7)	C113—H113	0.9500
C45—H45A	0.9900	C114—C115	1.465 (7)
C45—H45B	0.9900	C114—H114	0.9500
C46—C51	1.391 (7)	C115—C116	1.388 (7)
C46—C47	1.397 (7)	C115—C120	1.391 (8)
C47—C48	1.379 (7)	C116—C117	1.391 (7)
C47—H47	0.9500	C116—H116	0.9500
C48—C49	1.373 (8)	C117—C118	1.375 (8)
C48—H48	0.9500	C117—H117	0.9500
C49—C50	1.377 (8)	C118—C119	1.378 (8)
C49—H49	0.9500	C118—H118	0.9500
C50—C51	1.381 (7)	C119—C120	1.394 (8)
C50—H50	0.9500	C119—H119	0.9500
C51—H51	0.9500	C120—H120	0.9500
			
C8—Sn1—C1	120.6 (2)	C54—C53—C52	122.7 (5)
C8—Sn1—C15	118.4 (2)	C54—C53—H53	118.6
C1—Sn1—C15	119.9 (2)	C52—C53—H53	118.6
C8—Sn1—O1	94.91 (17)	C53—C54—C55	128.9 (5)
C1—Sn1—O1	94.35 (18)	C53—C54—H54	115.6
C15—Sn1—O1	90.95 (15)	C55—C54—H54	115.6
C8—Sn1—O2^i^	88.82 (17)	C60—C55—C56	118.2 (5)
C1—Sn1—O2^i^	90.56 (17)	C60—C55—C54	119.6 (5)
C15—Sn1—O2^i^	80.29 (17)	C56—C55—C54	122.2 (5)
O1—Sn1—O2^i^	171.2 (1)	C55—C56—C57	119.7 (6)
C45—Sn2—C38	120.1 (2)	C55—C56—H56	120.2
C45—Sn2—C31	118.6 (2)	C57—C56—H56	120.2
C38—Sn2—C31	119.9 (2)	C58—C57—C56	120.5 (6)
C45—Sn2—O3	95.81 (17)	C58—C57—H57	119.8
C38—Sn2—O3	95.27 (17)	C56—C57—H57	119.8
C31—Sn2—O3	90.46 (15)	C57—C58—C59	120.6 (6)
C45—Sn2—O4^ii^	89.23 (16)	C57—C58—H58	119.7
C38—Sn2—O4^ii^	90.00 (16)	C59—C58—H58	119.7
C31—Sn2—O4^ii^	79.16 (17)	C58—C59—C60	119.5 (6)
O3—Sn2—O4^ii^	169.6 (1)	C58—C59—H59	120.3
C68—Sn3—O5	97.63 (17)	C60—C59—H59	120.3
C68—Sn3—C75	120.36 (19)	C59—C60—C55	121.5 (6)
O5—Sn3—C75	98.06 (17)	C59—C60—H60	119.3
C68—Sn3—C61	119.5 (2)	C55—C60—H60	119.3
O5—Sn3—C61	91.71 (16)	C62—C61—Sn3	119.3 (3)
C75—Sn3—C61	117.1 (2)	C62—C61—H61A	107.5
C68—Sn3—O6^iii^	86.80 (17)	Sn3—C61—H61A	107.5
O5—Sn3—O6^iii^	168.4 (1)	C62—C61—H61B	107.5
C75—Sn3—O6^iii^	88.87 (16)	Sn3—C61—H61B	107.5
C61—Sn3—O6^iii^	76.76 (16)	H61A—C61—H61B	107.0
C105—Sn4—C98	119.4 (2)	C67—C62—C63	117.8 (5)
C105—Sn4—C91	119.1 (2)	C67—C62—C61	121.2 (5)
C98—Sn4—C91	120.1 (2)	C63—C62—C61	121.0 (5)
C105—Sn4—O7	90.16 (15)	C64—C63—C62	121.6 (5)
C98—Sn4—O7	96.21 (17)	C64—C63—H63	119.2
C91—Sn4—O7	95.52 (17)	C62—C63—H63	119.2
C105—Sn4—O8^iv^	79.21 (16)	C65—C64—C63	119.9 (5)
C98—Sn4—O8^iv^	89.56 (17)	C65—C64—H64	120.1
C91—Sn4—O8^iv^	89.27 (16)	C63—C64—H64	120.1
O7—Sn4—O8^iv^	169.4 (1)	C64—C65—C66	119.0 (5)
C22—O1—Sn1	122.0 (3)	C64—C65—H65	120.5
C22—O2—Sn1^v^	141.0 (3)	C66—C65—H65	120.5
C52—O3—Sn2	121.4 (3)	C67—C66—C65	120.9 (5)
C52—O4—Sn2^vi^	143.3 (3)	C67—C66—H66	119.6
C82—O5—Sn3	122.6 (3)	C65—C66—H66	119.6
C82—O6—Sn3^vii^	151.3 (3)	C62—C67—C66	120.8 (5)
C112—O7—Sn4	120.8 (3)	C62—C67—H67	119.6
C112—O8—Sn4^viii^	141.0 (3)	C66—C67—H67	119.6
C2—C1—Sn1	113.0 (4)	C69—C68—Sn3	113.5 (3)
C2—C1—H1A	109.0	C69—C68—H68A	108.9
Sn1—C1—H1A	109.0	Sn3—C68—H68A	108.9
C2—C1—H1B	109.0	C69—C68—H68B	108.9
Sn1—C1—H1B	109.0	Sn3—C68—H68B	108.9
H1A—C1—H1B	107.8	H68A—C68—H68B	107.7
C7—C2—C3	117.2 (6)	C74—C69—C70	118.4 (5)
C7—C2—C1	122.3 (5)	C74—C69—C68	121.5 (5)
C3—C2—C1	120.5 (6)	C70—C69—C68	120.0 (5)
C4—C3—C2	119.7 (6)	C71—C70—C69	121.0 (6)
C4—C3—H3	120.1	C71—C70—H70	119.5
C2—C3—H3	120.1	C69—C70—H70	119.5
C5—C4—C3	121.5 (6)	C72—C71—C70	120.3 (5)
C5—C4—H4	119.2	C72—C71—H71	119.9
C3—C4—H4	119.2	C70—C71—H71	119.9
C6—C5—C4	118.9 (6)	C71—C72—C73	120.0 (5)
C6—C5—H5	120.5	C71—C72—H72	120.0
C4—C5—H5	120.5	C73—C72—H72	120.0
C5—C6—C7	121.3 (6)	C72—C73—C74	119.7 (6)
C5—C6—H6	119.3	C72—C73—H73	120.2
C7—C6—H6	119.3	C74—C73—H73	120.2
C2—C7—C6	121.2 (6)	C69—C74—C73	120.6 (5)
C2—C7—H7	119.4	C69—C74—H74	119.7
C6—C7—H7	119.4	C73—C74—H74	119.7
C9—C8—Sn1	114.3 (4)	C76—C75—Sn3	113.1 (3)
C9—C8—H8A	108.7	C76—C75—H75A	109.0
Sn1—C8—H8A	108.7	Sn3—C75—H75A	109.0
C9—C8—H8B	108.7	C76—C75—H75B	109.0
Sn1—C8—H8B	108.7	Sn3—C75—H75B	109.0
H8A—C8—H8B	107.6	H75A—C75—H75B	107.8
C14—C9—C10	117.5 (6)	C81—C76—C77	117.1 (5)
C14—C9—C8	121.7 (5)	C81—C76—C75	121.6 (5)
C10—C9—C8	120.8 (5)	C77—C76—C75	121.3 (5)
C11—C10—C9	121.4 (6)	C78—C77—C76	121.0 (5)
C11—C10—H10	119.3	C78—C77—H77	119.5
C9—C10—H10	119.3	C76—C77—H77	119.5
C10—C11—C12	119.9 (6)	C79—C78—C77	119.6 (5)
C10—C11—H11	120.1	C79—C78—H78	120.2
C12—C11—H11	120.1	C77—C78—H78	120.2
C11—C12—C13	120.2 (6)	C78—C79—C80	121.2 (5)
C11—C12—H12	119.9	C78—C79—H79	119.4
C13—C12—H12	119.9	C80—C79—H79	119.4
C14—C13—C12	118.6 (6)	C79—C80—C81	118.9 (6)
C14—C13—H13	120.7	C79—C80—H80	120.6
C12—C13—H13	120.7	C81—C80—H80	120.6
C13—C14—C9	122.4 (6)	C80—C81—C76	122.2 (5)
C13—C14—H14	118.8	C80—C81—H81	118.9
C9—C14—H14	118.8	C76—C81—H81	118.9
C16—C15—Sn1	119.7 (4)	O6—C82—O5	123.2 (5)
C16—C15—H15A	107.4	O6—C82—C83	120.5 (5)
Sn1—C15—H15A	107.4	O5—C82—C83	116.3 (4)
C16—C15—H15B	107.4	C84—C83—C82	122.2 (5)
Sn1—C15—H15B	107.4	C84—C83—H83	118.9
H15A—C15—H15B	106.9	C82—C83—H83	118.9
C21—C16—C17	117.3 (5)	C83—C84—C85	128.7 (5)
C21—C16—C15	121.3 (5)	C83—C84—H84	115.6
C17—C16—C15	121.4 (5)	C85—C84—H84	115.6
C18—C17—C16	121.0 (5)	C86—C85—C90	117.9 (5)
C18—C17—H17	119.5	C86—C85—C84	118.0 (5)
C16—C17—H17	119.5	C90—C85—C84	124.0 (5)
C19—C18—C17	121.4 (5)	C87—C86—C85	121.9 (5)
C19—C18—H18	119.3	C87—C86—H86	119.0
C17—C18—H18	119.3	C85—C86—H86	119.0
C18—C19—C20	118.9 (5)	C86—C87—C88	119.2 (5)
C18—C19—H19	120.6	C86—C87—H87	120.4
C20—C19—H19	120.6	C88—C87—H87	120.4
C19—C20—C21	120.3 (6)	C87—C88—C89	120.3 (5)
C19—C20—H20	119.8	C87—C88—H88	119.8
C21—C20—H20	119.8	C89—C88—H88	119.8
C16—C21—C20	121.2 (5)	C90—C89—C88	120.2 (5)
C16—C21—H21	119.4	C90—C89—H89	119.9
C20—C21—H21	119.4	C88—C89—H89	119.9
O2—C22—O1	121.8 (5)	C89—C90—C85	120.4 (5)
O2—C22—C23	120.9 (5)	C89—C90—H90	119.8
O1—C22—C23	117.3 (5)	C85—C90—H90	119.8
C24—C23—C22	122.0 (5)	C92—C91—Sn4	111.6 (3)
C24—C23—H23	119.0	C92—C91—H91A	109.3
C22—C23—H23	119.0	Sn4—C91—H91A	109.3
C23—C24—C25	127.1 (5)	C92—C91—H91B	109.3
C23—C24—H24	116.5	Sn4—C91—H91B	109.3
C25—C24—H24	116.5	H91A—C91—H91B	108.0
C30—C25—C26	117.3 (5)	C93—C92—C97	117.7 (5)
C30—C25—C24	123.3 (5)	C93—C92—C91	120.8 (5)
C26—C25—C24	119.4 (4)	C97—C92—C91	121.4 (5)
C27—C26—C25	121.3 (5)	C92—C93—C94	122.2 (6)
C27—C26—H26	119.3	C92—C93—H93	118.9
C25—C26—H26	119.3	C94—C93—H93	118.9
C28—C27—C26	119.6 (5)	C93—C94—C95	120.0 (5)
C28—C27—H27	120.2	C93—C94—H94	120.0
C26—C27—H27	120.2	C95—C94—H94	120.0
C29—C28—C27	120.3 (5)	C94—C95—C96	119.7 (5)
C29—C28—H28	119.9	C94—C95—H95	120.1
C27—C28—H28	119.9	C96—C95—H95	120.1
C28—C29—C30	120.1 (5)	C95—C96—C97	119.6 (5)
C28—C29—H29	120.0	C95—C96—H96	120.2
C30—C29—H29	120.0	C97—C96—H96	120.2
C29—C30—C25	121.3 (5)	C96—C97—C92	120.8 (5)
C29—C30—H30	119.3	C96—C97—H97	119.6
C25—C30—H30	119.3	C92—C97—H97	119.6
C32—C31—Sn2	117.1 (3)	C99—C98—Sn4	113.4 (3)
C32—C31—H31A	108.0	C99—C98—H98A	108.9
Sn2—C31—H31A	108.0	Sn4—C98—H98A	108.9
C32—C31—H31B	108.0	C99—C98—H98B	108.9
Sn2—C31—H31B	108.0	Sn4—C98—H98B	108.9
H31A—C31—H31B	107.3	H98A—C98—H98B	107.7
C37—C32—C33	116.3 (5)	C104—C99—C100	118.2 (5)
C37—C32—C31	121.9 (5)	C104—C99—C98	120.2 (5)
C33—C32—C31	121.8 (5)	C100—C99—C98	121.6 (5)
C34—C33—C32	121.9 (5)	C101—C100—C99	119.6 (6)
C34—C33—H33	119.0	C101—C100—H100	120.2
C32—C33—H33	119.0	C99—C100—H100	120.2
C33—C34—C35	120.4 (5)	C102—C101—C100	121.5 (6)
C33—C34—H34	119.8	C102—C101—H101	119.3
C35—C34—H34	119.8	C100—C101—H101	119.3
C34—C35—C36	119.2 (5)	C103—C102—C101	119.0 (6)
C34—C35—H35	120.4	C103—C102—H102	120.5
C36—C35—H35	120.4	C101—C102—H102	120.5
C37—C36—C35	119.5 (5)	C102—C103—C104	120.9 (6)
C37—C36—H36	120.3	C102—C103—H103	119.5
C35—C36—H36	120.3	C104—C103—H103	119.5
C36—C37—C32	122.6 (5)	C99—C104—C103	120.7 (5)
C36—C37—H37	118.7	C99—C104—H104	119.7
C32—C37—H37	118.7	C103—C104—H104	119.7
C39—C38—Sn2	114.5 (3)	C106—C105—Sn4	118.8 (3)
C39—C38—H38A	108.6	C106—C105—H10A	107.6
Sn2—C38—H38A	108.6	Sn4—C105—H10A	107.6
C39—C38—H38B	108.6	C106—C105—H10B	107.6
Sn2—C38—H38B	108.6	Sn4—C105—H10B	107.6
H38A—C38—H38B	107.6	H10A—C105—H10B	107.1
C44—C39—C40	116.8 (5)	C111—C106—C107	117.1 (5)
C44—C39—C38	122.8 (5)	C111—C106—C105	121.5 (5)
C40—C39—C38	120.4 (4)	C107—C106—C105	121.4 (5)
C41—C40—C39	121.7 (5)	C108—C107—C106	122.0 (5)
C41—C40—H40	119.1	C108—C107—H107	119.0
C39—C40—H40	119.1	C106—C107—H107	119.0
C42—C41—C40	120.2 (5)	C107—C108—C109	118.9 (5)
C42—C41—H41	119.9	C107—C108—H108	120.5
C40—C41—H41	119.9	C109—C108—H108	120.5
C43—C42—C41	119.4 (5)	C110—C109—C108	120.4 (5)
C43—C42—H42	120.3	C110—C109—H109	119.8
C41—C42—H42	120.3	C108—C109—H109	119.8
C42—C43—C44	120.2 (5)	C109—C110—C111	119.9 (6)
C42—C43—H43	119.9	C109—C110—H110	120.1
C44—C43—H43	119.9	C111—C110—H110	120.1
C39—C44—C43	121.6 (5)	C110—C111—C106	121.8 (6)
C39—C44—H44	119.2	C110—C111—H111	119.1
C43—C44—H44	119.2	C106—C111—H111	119.1
C46—C45—Sn2	113.8 (3)	O8—C112—O7	122.3 (4)
C46—C45—H45A	108.8	O8—C112—C113	121.3 (5)
Sn2—C45—H45A	108.8	O7—C112—C113	116.4 (4)
C46—C45—H45B	108.8	C114—C113—C112	122.5 (5)
Sn2—C45—H45B	108.8	C114—C113—H113	118.8
H45A—C45—H45B	107.7	C112—C113—H113	118.8
C51—C46—C47	117.3 (5)	C113—C114—C115	127.6 (5)
C51—C46—C45	122.1 (5)	C113—C114—H114	116.2
C47—C46—C45	120.5 (5)	C115—C114—H114	116.2
C48—C47—C46	121.0 (5)	C116—C115—C120	117.8 (5)
C48—C47—H47	119.5	C116—C115—C114	120.2 (5)
C46—C47—H47	119.5	C120—C115—C114	122.0 (5)
C49—C48—C47	120.9 (6)	C115—C116—C117	121.2 (5)
C49—C48—H48	119.5	C115—C116—H116	119.4
C47—C48—H48	119.5	C117—C116—H116	119.4
C48—C49—C50	119.0 (5)	C118—C117—C116	120.1 (5)
C48—C49—H49	120.5	C118—C117—H117	120.0
C50—C49—H49	120.5	C116—C117—H117	120.0
C49—C50—C51	120.6 (5)	C117—C118—C119	120.0 (5)
C49—C50—H50	119.7	C117—C118—H118	120.0
C51—C50—H50	119.7	C119—C118—H118	120.0
C50—C51—C46	121.2 (5)	C118—C119—C120	119.7 (5)
C50—C51—H51	119.4	C118—C119—H119	120.1
C46—C51—H51	119.4	C120—C119—H119	120.1
O4—C52—O3	122.8 (5)	C115—C120—C119	121.2 (5)
O4—C52—C53	120.8 (5)	C115—C120—H120	119.4
O3—C52—C53	116.4 (4)	C119—C120—H120	119.4
			
C8—Sn1—O1—C22	−57.0 (4)	C55—C56—C57—C58	−0.1 (8)
C1—Sn1—O1—C22	64.3 (4)	C56—C57—C58—C59	0.5 (9)
C15—Sn1—O1—C22	−175.6 (4)	C57—C58—C59—C60	−1.3 (9)
C45—Sn2—O3—C52	−60.5 (4)	C58—C59—C60—C55	1.6 (9)
C38—Sn2—O3—C52	60.6 (4)	C56—C55—C60—C59	−1.2 (8)
C31—Sn2—O3—C52	−179.3 (4)	C54—C55—C60—C59	178.5 (5)
C68—Sn3—O5—C82	56.0 (4)	C68—Sn3—C61—C62	92.7 (5)
C75—Sn3—O5—C82	−66.3 (4)	O5—Sn3—C61—C62	−7.0 (4)
C61—Sn3—O5—C82	176.1 (4)	C75—Sn3—C61—C62	−107.0 (4)
O6^iii^—Sn3—O5—C82	167.8 (6)	O6^iii^—Sn3—C61—C62	171.3 (5)
C105—Sn4—O7—C112	177.4 (4)	Sn3—C61—C62—C67	−103.0 (5)
C98—Sn4—O7—C112	57.7 (4)	Sn3—C61—C62—C63	77.6 (6)
C91—Sn4—O7—C112	−63.4 (4)	C67—C62—C63—C64	−0.2 (8)
C8—Sn1—C1—C2	−141.8 (4)	C61—C62—C63—C64	179.2 (5)
C15—Sn1—C1—C2	26.3 (5)	C62—C63—C64—C65	0.7 (8)
O1—Sn1—C1—C2	119.9 (4)	C63—C64—C65—C66	−1.6 (8)
O2^i^—Sn1—C1—C2	−52.8 (4)	C64—C65—C66—C67	2.0 (9)
Sn1—C1—C2—C7	110.4 (5)	C63—C62—C67—C66	0.5 (8)
Sn1—C1—C2—C3	−67.2 (6)	C61—C62—C67—C66	−178.8 (5)
C7—C2—C3—C4	−1.3 (9)	C65—C66—C67—C62	−1.5 (9)
C1—C2—C3—C4	176.4 (6)	O5—Sn3—C68—C69	104.2 (4)
C2—C3—C4—C5	2.6 (11)	C75—Sn3—C68—C69	−151.7 (3)
C3—C4—C5—C6	−1.1 (11)	C61—Sn3—C68—C69	7.9 (4)
C4—C5—C6—C7	−1.7 (10)	O6^iii^—Sn3—C68—C69	−64.9 (4)
C3—C2—C7—C6	−1.5 (9)	Sn3—C68—C69—C74	115.9 (5)
C1—C2—C7—C6	−179.2 (5)	Sn3—C68—C69—C70	−66.9 (5)
C5—C6—C7—C2	3.1 (10)	C74—C69—C70—C71	1.5 (8)
C1—Sn1—C8—C9	147.6 (4)	C68—C69—C70—C71	−175.8 (5)
C15—Sn1—C8—C9	−20.7 (5)	C69—C70—C71—C72	−1.2 (9)
O1—Sn1—C8—C9	−114.4 (4)	C70—C71—C72—C73	0.5 (9)
O2^i^—Sn1—C8—C9	57.6 (4)	C71—C72—C73—C74	−0.1 (9)
Sn1—C8—C9—C14	−112.0 (5)	C70—C69—C74—C73	−1.1 (8)
Sn1—C8—C9—C10	69.1 (6)	C68—C69—C74—C73	176.1 (5)
C14—C9—C10—C11	−0.1 (9)	C72—C73—C74—C69	0.5 (9)
C8—C9—C10—C11	178.8 (5)	C68—Sn3—C75—C76	139.7 (4)
C9—C10—C11—C12	−0.5 (9)	O5—Sn3—C75—C76	−116.5 (4)
C10—C11—C12—C13	0.9 (10)	C61—Sn3—C75—C76	−20.4 (5)
C11—C12—C13—C14	−0.5 (11)	O6^iii^—Sn3—C75—C76	54.1 (4)
C12—C13—C14—C9	−0.2 (11)	Sn3—C75—C76—C81	69.9 (6)
C10—C9—C14—C13	0.5 (10)	Sn3—C75—C76—C77	−108.4 (5)
C8—C9—C14—C13	−178.4 (6)	C81—C76—C77—C78	−0.8 (7)
C8—Sn1—C15—C16	−112.9 (4)	C75—C76—C77—C78	177.6 (5)
C1—Sn1—C15—C16	78.8 (5)	C76—C77—C78—C79	−1.0 (8)
O1—Sn1—C15—C16	−16.8 (4)	C77—C78—C79—C80	2.6 (8)
O2^i^—Sn1—C15—C16	163.8 (5)	C78—C79—C80—C81	−2.4 (8)
Sn1—C15—C16—C21	−103.6 (5)	C79—C80—C81—C76	0.5 (8)
Sn1—C15—C16—C17	76.8 (6)	C77—C76—C81—C80	1.1 (8)
C21—C16—C17—C18	−0.8 (8)	C75—C76—C81—C80	−177.3 (5)
C15—C16—C17—C18	178.8 (5)	Sn3^vii^—O6—C82—O5	176.3 (4)
C16—C17—C18—C19	2.0 (8)	Sn3^vii^—O6—C82—C83	−2.7 (10)
C17—C18—C19—C20	−2.2 (8)	Sn3—O5—C82—O6	2.5 (7)
C18—C19—C20—C21	1.4 (9)	Sn3—O5—C82—C83	−178.5 (3)
C17—C16—C21—C20	0.0 (8)	O6—C82—C83—C84	179.6 (5)
C15—C16—C21—C20	−179.6 (5)	O5—C82—C83—C84	0.6 (8)
C19—C20—C21—C16	−0.3 (9)	C82—C83—C84—C85	−179.7 (5)
Sn1^v^—O2—C22—O1	−177.8 (4)	C83—C84—C85—C86	166.8 (5)
Sn1^v^—O2—C22—C23	1.7 (9)	C83—C84—C85—C90	−11.2 (9)
Sn1—O1—C22—O2	−2.3 (7)	C90—C85—C86—C87	1.2 (8)
Sn1—O1—C22—C23	178.2 (4)	C84—C85—C86—C87	−176.9 (5)
O2—C22—C23—C24	−177.4 (5)	C85—C86—C87—C88	−1.8 (8)
O1—C22—C23—C24	2.1 (8)	C86—C87—C88—C89	0.3 (8)
C22—C23—C24—C25	178.9 (5)	C87—C88—C89—C90	1.7 (8)
C23—C24—C25—C30	8.2 (9)	C88—C89—C90—C85	−2.3 (8)
C23—C24—C25—C26	−171.8 (5)	C86—C85—C90—C89	0.8 (8)
C30—C25—C26—C27	0.8 (9)	C84—C85—C90—C89	178.8 (5)
C24—C25—C26—C27	−179.2 (5)	C105—Sn4—C91—C92	−13.6 (4)
C25—C26—C27—C28	0.0 (9)	C98—Sn4—C91—C92	152.7 (3)
C26—C27—C28—C29	−0.9 (9)	O7—Sn4—C91—C92	−106.8 (4)
C27—C28—C29—C30	0.9 (9)	O8^iv^—Sn4—C91—C92	63.6 (4)
C28—C29—C30—C25	0.0 (9)	Sn4—C91—C92—C93	68.4 (6)
C26—C25—C30—C29	−0.8 (9)	Sn4—C91—C92—C97	−109.9 (5)
C24—C25—C30—C29	179.2 (5)	C97—C92—C93—C94	0.7 (9)
C45—Sn2—C31—C32	−103.1 (4)	C91—C92—C93—C94	−177.6 (6)
C38—Sn2—C31—C32	90.1 (5)	C92—C93—C94—C95	0.5 (10)
O3—Sn2—C31—C32	−6.3 (4)	C93—C94—C95—C96	−1.6 (10)
O4^ii^—Sn2—C31—C32	173.8 (4)	C94—C95—C96—C97	1.5 (9)
Sn2—C31—C32—C37	−90.5 (5)	C95—C96—C97—C92	−0.4 (9)
Sn2—C31—C32—C33	87.8 (6)	C93—C92—C97—C96	−0.7 (8)
C37—C32—C33—C34	1.6 (8)	C91—C92—C97—C96	177.6 (5)
C31—C32—C33—C34	−176.8 (5)	C105—Sn4—C98—C99	23.6 (5)
C32—C33—C34—C35	−1.6 (9)	C91—Sn4—C98—C99	−142.7 (4)
C33—C34—C35—C36	0.3 (9)	O7—Sn4—C98—C99	117.3 (4)
C34—C35—C36—C37	1.0 (8)	O8^iv^—Sn4—C98—C99	−53.7 (4)
C35—C36—C37—C32	−1.0 (8)	Sn4—C98—C99—C104	−69.2 (6)
C33—C32—C37—C36	−0.3 (7)	Sn4—C98—C99—C100	110.2 (5)
C31—C32—C37—C36	178.1 (5)	C104—C99—C100—C101	−0.4 (8)
C45—Sn2—C38—C39	−137.4 (4)	C98—C99—C100—C101	−179.9 (5)
C31—Sn2—C38—C39	29.1 (5)	C99—C100—C101—C102	2.1 (10)
O3—Sn2—C38—C39	122.7 (4)	C100—C101—C102—C103	−3.1 (10)
O4^ii^—Sn2—C38—C39	−48.3 (4)	C101—C102—C103—C104	2.5 (9)
Sn2—C38—C39—C44	110.5 (5)	C100—C99—C104—C103	−0.1 (8)
Sn2—C38—C39—C40	−69.8 (6)	C98—C99—C104—C103	179.3 (5)
C44—C39—C40—C41	−2.0 (7)	C102—C103—C104—C99	−0.9 (9)
C38—C39—C40—C41	178.3 (5)	C98—Sn4—C105—C106	103.1 (4)
C39—C40—C41—C42	1.6 (8)	C91—Sn4—C105—C106	−90.5 (4)
C40—C41—C42—C43	−0.3 (8)	O7—Sn4—C105—C106	5.9 (4)
C41—C42—C43—C44	−0.4 (8)	O8^iv^—Sn4—C105—C106	−173.6 (4)
C40—C39—C44—C43	1.2 (7)	Sn4—C105—C106—C111	101.6 (5)
C38—C39—C44—C43	−179.1 (5)	Sn4—C105—C106—C107	−78.2 (6)
C42—C43—C44—C39	−0.1 (8)	C111—C106—C107—C108	0.2 (7)
C38—Sn2—C45—C46	153.9 (3)	C105—C106—C107—C108	−179.9 (5)
C31—Sn2—C45—C46	−12.8 (4)	C106—C107—C108—C109	−1.0 (8)
O3—Sn2—C45—C46	−106.6 (4)	C107—C108—C109—C110	0.8 (8)
O4^ii^—Sn2—C45—C46	64.4 (4)	C108—C109—C110—C111	0.2 (9)
Sn2—C45—C46—C51	−109.2 (5)	C109—C110—C111—C106	−1.0 (8)
Sn2—C45—C46—C47	73.5 (5)	C107—C106—C111—C110	0.8 (8)
C51—C46—C47—C48	−0.3 (8)	C105—C106—C111—C110	−179.1 (5)
C45—C46—C47—C48	177.1 (5)	Sn4^viii^—O8—C112—O7	178.0 (3)
C46—C47—C48—C49	−0.9 (9)	Sn4^viii^—O8—C112—C113	−2.8 (8)
C47—C48—C49—C50	1.9 (9)	Sn4—O7—C112—O8	2.1 (7)
C48—C49—C50—C51	−1.7 (9)	Sn4—O7—C112—C113	−177.1 (3)
C49—C50—C51—C46	0.5 (9)	O8—C112—C113—C114	−178.3 (5)
C47—C46—C51—C50	0.5 (8)	O7—C112—C113—C114	0.9 (8)
C45—C46—C51—C50	−176.9 (5)	C112—C113—C114—C115	177.3 (5)
Sn2^vi^—O4—C52—O3	−180.0 (3)	C113—C114—C115—C116	−171.6 (6)
Sn2^vi^—O4—C52—C53	0.3 (8)	C113—C114—C115—C120	6.2 (9)
Sn2—O3—C52—O4	2.2 (6)	C120—C115—C116—C117	−1.3 (9)
Sn2—O3—C52—C53	−178.1 (3)	C114—C115—C116—C117	176.7 (5)
O4—C52—C53—C54	−170.9 (5)	C115—C116—C117—C118	1.0 (9)
O3—C52—C53—C54	9.3 (7)	C116—C117—C118—C119	−0.5 (9)
C52—C53—C54—C55	179.2 (5)	C117—C118—C119—C120	0.2 (10)
C53—C54—C55—C60	−169.4 (5)	C116—C115—C120—C119	1.0 (10)
C53—C54—C55—C56	10.3 (9)	C114—C115—C120—C119	−176.9 (6)
C60—C55—C56—C57	0.4 (8)	C118—C119—C120—C115	−0.5 (10)
C54—C55—C56—C57	−179.2 (5)		

Symmetry codes: (i) −*x*+1, *y*−1/2, −*z*; (ii) −*x*+1, *y*−1/2, −*z*+1; (iii) −*x*, *y*+1/2, −*z*+1; (iv) −*x*, *y*+1/2, −*z*+2; (v) −*x*+1, *y*+1/2, −*z*; (vi) −*x*+1, *y*+1/2, −*z*+1; (vii) −*x*, *y*−1/2, −*z*+1; (viii) −*x*, *y*−1/2, −*z*+2.
